# A new approach to chromosome-wide analysis of X-linked markers identifies new associations in Asian and European case-parent triads of orofacial clefts

**DOI:** 10.1371/journal.pone.0183772

**Published:** 2017-09-06

**Authors:** Øivind Skare, Håkon K. Gjessing, Miriam Gjerdevik, Øystein A. Haaland, Julia Romanowska, Rolv T. Lie, Astanand Jugessur

**Affiliations:** 1 Department of Occupational Medicine and Epidemiology, National Institute of Occupational Health, Oslo, Norway; 2 Centre for Fertility and Health (CeFH), Norwegian Institute of Public Health, Oslo, Norway; 3 Department of Global Public Health and Primary Care, University of Bergen, Bergen, Norway; 4 Department of Genetic Research and Bioinformatics, Norwegian Institute of Public Health, Oslo, Norway; 5 Centre for Burden of Disease, Norwegian Institute of Public Health, Oslo, Norway; 6 Computational Biology Unit, University of Bergen, Bergen, Norway; 7 Department of Health Registries, Norwegian Institute of Public Health, Oslo, Norway; Medical University of South Carolina, UNITED STATES

## Abstract

**Background:**

GWAS discoveries on the X-chromosome are underrepresented in the literature primarily because the analytical tools that have been applied were originally designed for autosomal markers. Our objective here is to employ a new robust and flexible tool for chromosome-wide analysis of X-linked markers in complex traits. Orofacial clefts are good candidates for such analysis because of the consistently observed excess of females with cleft palate only (CPO) and excess of males with cleft lip with or without cleft palate (CL/P).

**Methods:**

Genotypes for 14,486 X-chromosome SNPs in 1,291 Asian and 1,118 European isolated cleft triads were available from a previously published GWAS. The R-package HAPLIN enables genome-wide–level analyses as well as statistical power simulations for a range of biologic scenarios. We analyzed isolated CL/P and isolated CPO for each ethnicity in HAPLIN, using a sliding-window approach to haplotype analysis and two different statistical models, with and without X-inactivation in females.

**Results:**

There was a larger number of associations in the Asian versus the European sample, and similar to previous reports that have analyzed the same GWAS dataset using different methods, we identified associations with *EFNB1/PJA1* and *DMD*. In addition, new associations were detected with several other genes, among which *KLHL4*, *TBX22*, *CPXCR1* and *BCOR* were noteworthy because of their roles in clefting syndromes. A few of the associations were only detected by one particular X-inactivation model, whereas a few others were only detected in one sex.

**Discussion/Conclusion:**

We found new support for the involvement of X-linked variants in isolated clefts. The associations were specific for ethnicity, sex and model parameterization, highlighting the need for flexible tools that are capable of detecting and estimating such effects. Further efforts are needed to verify and elucidate the potential roles of *EFNB1/PJA1*, *KLHL4*, *TBX22*, *CPXCR1* and *BCOR* in isolated clefts.

## Introduction

Genome-wide association studies (GWAS) have unraveled many promising genes and loci for isolated clefts [1–4], but with few exceptions [5–9], most of these discoveries have been on autosomes. This is primarily because the methods that have been applied were originally designed for autosomal markers. With growing evidence pointing to a role for X-linked variants in several complex traits [10], including cancer, Type 1 and Type 2 diabetes, male-pattern baldness, human height, and primary tooth development, methods that can also handle X-chromosome markers have started to appear in the literature (reviewed in [5]). These methods are particularly useful for studying traits in which there is a skewed sex ratio in prevalence and traits that are characterized by sexual dimorphism. Examples include orofacial clefts, where males have a higher prevalence of cleft lip with or without cleft palate (CL/P) and females a higher prevalence of cleft palate only (CPO), autism spectrum disorders where males are four times as likely to be affected as females, and anthropometric traits such as human height that exhibit sexual dimorphism [11, 12].

Searching for genetic associations between X-linked SNPs and isolated orofacial clefts is justified on several grounds. First, many of the genes associated with syndromic forms of clefting are located on the X-chromosome; e.g. ‘T-BOX 22’ (*TBX22*, Xq21.1), Anosmin 1 (*ANOS1*, formerly *KAL1*, Xp22.31), ‘Midline 1’ (*MID1*, Xp22), ‘PHD finger protein 8’ (*PHD8*, Xp11.22), ‘RNA-binding motif protein 10’ (*RBM10*, Xp11.23), and ‘Oral-facial-digital syndrome 1’ (*OFD1*, Xp22) (see [5] and references therein). It is plausible that these genes might also contribute to isolated clefts. ‘Interferon regulatory factor 6’ (*IRF6*) on chromosome 1q32.2 is an example of a gene that was first reported to cause a clefting syndrome (Van der Woude syndrome) and that was later shown to be involved in isolated clefts [13]. Second, despite the strong connections between X-linked genes and syndromic clefts, and the consistently observed skewed sex ratio in CPO and CL/P, only four chromosome-wide searches for associations have thus far been conducted for X-linked SNPs and isolated clefts [6–9]. Third, because the majority of GWAS have traditionally excluded non-autosomal SNPs prior to analysis, the development of methods for analyzing X-linked SNPs has lagged behind its autosomal counterpart [10]. Lastly, most methods for analyzing X-linked SNPs are derivatives of the transmission-disequilibrium test (TDT), which provide p-values for hypothesis-testing but not relative risks with confidence intervals that are more informative when interpreting disease risk across different studies and study-designs. We are aware of only five methods that provide such estimates: HAPLIN [5], UNPHASED [14], ‘Likelihood ratio test of association for X-linked markers’ (X-LRT) [15], ‘Parent-informed likelihood ratio test for the X-chromosome’ (PIX-LRT) [7], and a recent extension of PIX-LRT that enables haplotype analysis (PIX-HAP) [9].

With these gaps in mind, we previously published a comprehensive biostatistical toolkit for analyzing candidate genes on the X-chromosome under a set of causal scenarios relevant to an X-linked disease locus [5]. Our methods are flexible in that they can accommodate a wide variety of biologic scenarios, such as sex-specific baseline risk and X-inactivation in females. They can also estimate relative risks with confidence intervals for a given risk allele or haplotype [5]. In this paper, we develop these methods further to enable genome-wide–level analyses in our R-package HAPLIN [16] and search for associations between isolated clefts and X-linked SNPs from a previously published GWAS that included Norwegian cleft triads [17]. Three other reports have tested for associations in the same GWAS dataset using different statistical methods [6, 7, 9]. We repeat and extend these analyses using HAPLIN, both with and without the assumption of X-inactivation, with a sliding-window haplotype-based approach, and provide estimates of the discovered effects.

## Materials and methods

### Study populations

Genotypes for 14,486 SNPs in 1,291 Asian and 1,118 European case-parent triads of isolated orofacial clefts were available from a previously published GWAS on clefts that included Norwegian triads [6, 17]. These triads were recruited as part of an international cleft consortium comprising seven Asian and six European/US recruitment sites. Characteristics of the study populations, genotyping platform, and initial quality-control criteria used for data-cleaning have been reported elsewhere [6, 17, 18]. In brief, genotyping was performed on an Illumina Human610-Quad^®^ platform by the Center for Inherited Disease Research (CIDR) at the Johns Hopkins University, Baltimore, Maryland, USA. Genotypes for 589,945 SNPs (99.56% of the attempted SNPs) were released and subsequently deposited in the Database of Genotypes and Phenotypes (dbGaP; http://www.ncbi.nlm.nih.gov/gap), under study accession ID phs000094.v1.p1. Here, we used PLINK [19] for additional data-cleaning and excluded individuals with more than 10% missing genotypes, SNPs with more than 1% missing genotypes, and SNPs with a minor allele frequency (MAF) below 0.01. This left a total of 13,180 SNPs on the X-chromosome for the current analyses.

We combined the seven Asian populations into a single group which we refer to as the ‘Asian sample’ throughout this paper. Similarly, the six European/US populations were merged into a single group and referred to as the ‘European sample’. This pooling by ethnicity is justified because the case-parent triad design protects against population stratification [20, 21]. We analyzed isolated CPO and isolated CL/P in the Asian and European triads, respectively. Table 1 shows the distribution of triads by cleft category, ethnicity, and child’s sex.

**Table 1:** Number of isolated case-parent triads by cleft category, ethnicity and sex of child.

		No. of case-parent triads		
Ethnicity	Isolated cleft category	Males	Females	Total
Asian	CLO+CLP+CPO	781	510	1291
Asian	CLO	138	108	246
Asian	CL/P	681	357	1038
Asian	CLP	543	249	792
Asian	CPO	100	153	253
European	CLO+CLP+CPO	667	451	1118
European	CLO	176	116	292
European	CL/P	536	304	840
European	CLP	360	188	548
European	CPO	131	147	278

### Statistical methods

#### R-package HAPLIN

HAPLIN version 6.0.1 [16] was used to analyze one SNP at a time in single-marker analyses, and a maximum of four consecutive SNPs per window in sliding-window haplotype analyses. HAPLIN fits a log-linear model to genotype data from family triads and case-control collections. For more statistical power and flexibility, HAPLIN accommodates a special hybrid configuration in which case-parent triads and control-parent triads can be analyzed together [22]. HAPLIN implements a maximum likelihood model to make full use of the available genotype data, and uses the expectation-maximization (EM) algorithm to “impute” missing genotypes. This may happen if a SNP fails to be genotyped in a particular individual or an individual does not participate in the study. For the missing genotypes, HAPLIN computes a probability distribution of the possible missing genotypes to obtain correct standard errors, confidence intervals and likelihood ratio tests (LRT) for a given statistical model.

#### Sliding-window haplotype analysis in HAPLIN

HAPLIN reconstructs haplotypes from multiple SNPs and estimates relative risks with confidence intervals for a single copy (‘single dose’) or two copies (‘double dose’) of a target haplotype. We used the haplinSlide function in HAPLIN to analyze a sequence of overlapping windows of haplotypes consisting of up to four consecutive SNPs per window. The advantage of this approach is that it can potentially enclose a hitherto unidentified causal variant by having one or more genotyped SNPs flank it on either side. Because it is not obvious *a priori* whether analyzing SNPs individually would be more effective at detecting an association than haplotypes, both single-marker and sliding-window haplotype analyses were performed on the current GWAS dataset.

To handle the typically large data files generated by a genome-wide scan, HAPLIN currently employs the data storage format of the R-package GenABEL [23], and extends it to triad data provided in a standard pedigree file created by, for instance, PLINK [19]. For detailed information on data format and conversion, see http://people.uib.no/gjessing/genetics/software/haplin and the R help pages for HAPLIN.

#### Models for analyzing X-chromosome SNPs in HAPLIN

**Fig 1:**
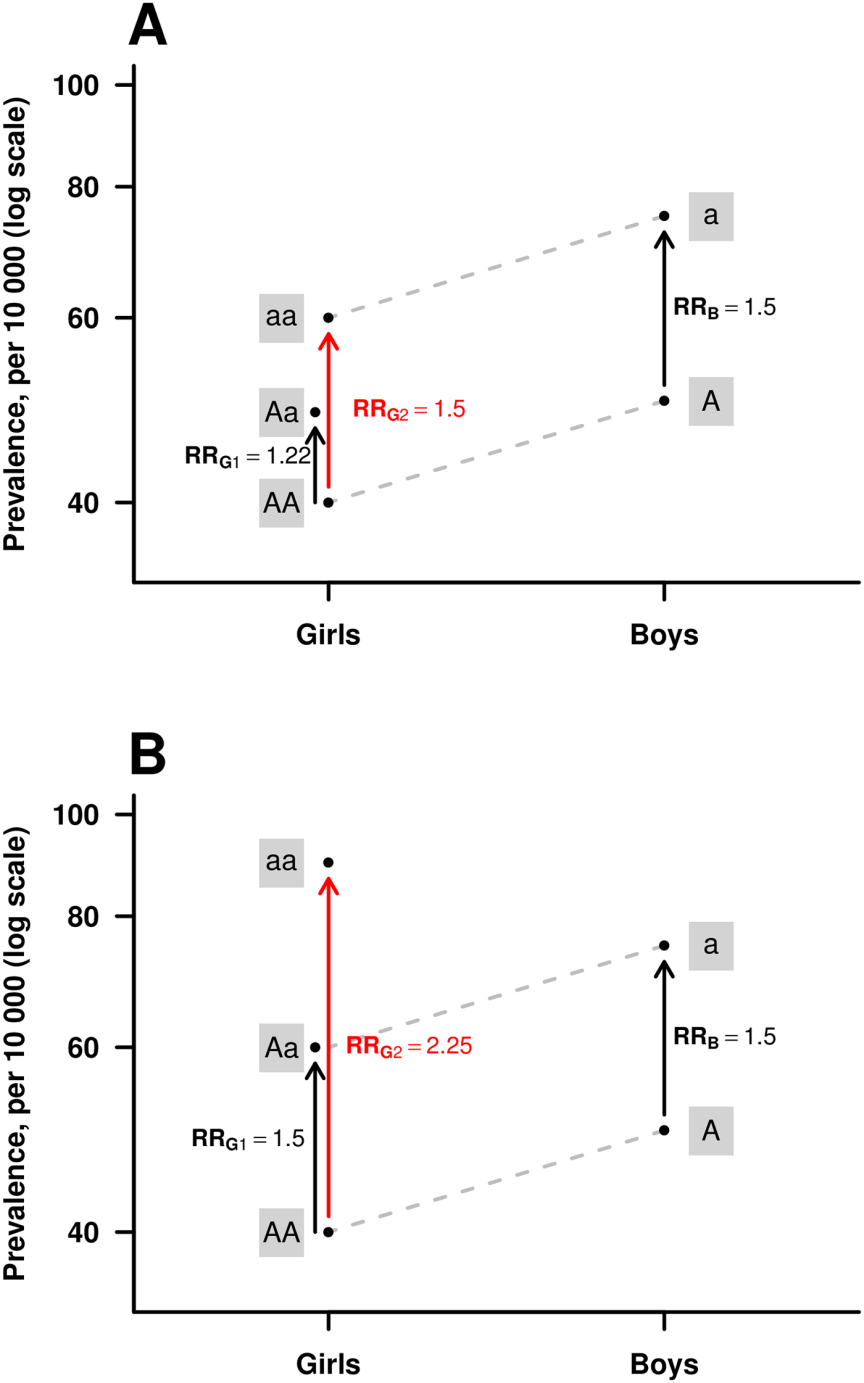
An illustration of the HAPLIN model for the X-chromosome. The red arrows show the relative risks (RR) associated with girls inheriting a double dose of the risk allele “a”, RRG2. Under the multiplicative risk model illustrated here, RRG2 = RRG1·RRG1, where RRG1 is the relative risk associated with girls inheriting a single dose of “a”. Under the assumption of X-inactivation (**Panel A**), the risk increase for girls inheriting a double dose of “a” is no larger than the increase for boys (RRB) inheriting the “a” allele, i.e. RRG2 = RRB. Under the assumption of no X-inactivation (**Panel B**), the risk increase for girls inheriting a single dose of “a” is the same as the increase for boys when inheriting the “a”, i.e. RRG1 = RRB, whereas the increase for girls inheriting a double dose is larger. The model allows different baseline prevalences for girls versus boys, here 40 versus 50 per 10 000, respectively.

HAPLIN caters to a range of causal scenarios—i.e., penetrance models—for an X-linked disease locus, conditional upon the assumptions made about allele-effects in males versus females [5, 24]. For instance, HAPLIN allows for sex-specific baseline risks, which are especially relevant for orofacial clefts given CL/P is more prevalent in males and CPO is more prevalent in females. It also allows for X-inactivation in females. All these models have been thoroughly described in our previous work ([5], Table 3). Here, we ran i) analyses with and without X-inactivation in females, and ii) separate analyses for males and females. In the analyses with X-inactivation, the relative risk for males is assumed to be equal to the relative risk for a double dose of the variant allele in females (Fig 1, Panel A). In the analyses without X-inactivation, the relative risk for males is assumed to be equal to the relative risk for a single dose of the variant allele in females (Fig 1, Panel B).

While the choice of model relates directly to a biologic interpretation, the combined-sex analyses use the data in a more comprehensive fashion and would thus be expected to provide more power than stratified models under appropriate settings. We ran a selection of power simulations to compare the performance of the models. All the models used in our GWAS scan assume a multiplicative dose-response relationship in females to enhance statistical power.

#### Post-processing of results

After the HAPLIN GWAS-run, groups of results were displayed in Manhattan plots, with the negative log_10_ of the observed p-values on one axis and the SNP positions on the other axis. To control for multiple testing, we applied a false discovery rate (FDR) method in which the original p-values are replaced by q-values [25]. The lower the q-value, the less likely an observed association is a false positive. We used a q-value ≤0.1 to assess statistical significance, which corresponds to an FDR of 10% among the *significant* SNPs. This method thus controls for the proportion of falsely rejected hypotheses among the rejected hypotheses (for more details, see [5]).

### Electronic database information

HAPLIN is implemented as a standard package in the R statistical software [26] and can be installed from the official R package archive, CRAN (https://cran.r-project.org). Our web site, http://people.uib.no/gjessing/genetics/software/haplin, provides further information.

### Ethics approval

Ethics approvals for the International Cleft Consortium were obtained from the respective institutional review boards of the participating sites. The consortium was formed in 2007 and each participating institution approved research protocols for the recruitment of case-parent triads from 13 individual sites. The participating sites included institutions in the US (Johns Hopkins University; University of Iowa; Utah State University; National Institute of Environmental Health Sciences (NIEHS); University of Pittsburgh), Denmark (University of Southern Denmark), Norway (University of Bergen), China (Peking University Health Science Center; Wuhan University; Peking Union Medical College; West China School of Stomatology, Sichuan University; School of Stomatology, Beijing University), Korea (Yonsei University), Taiwan (Chang Gung Memorial Hospital), and Singapore (KK Women’s & Children’s Hospital; National University of Singapore). For additional details on the recruitment sites, the research approvals and protocols, see the online ‘Supplementary Note’ of the original publication [17] as well as the study outline at dbGAP (https://www.ncbi.nlm.nih.gov/gap) under study accession number phs000094.v1.p1.

## Results

**Fig 2:**
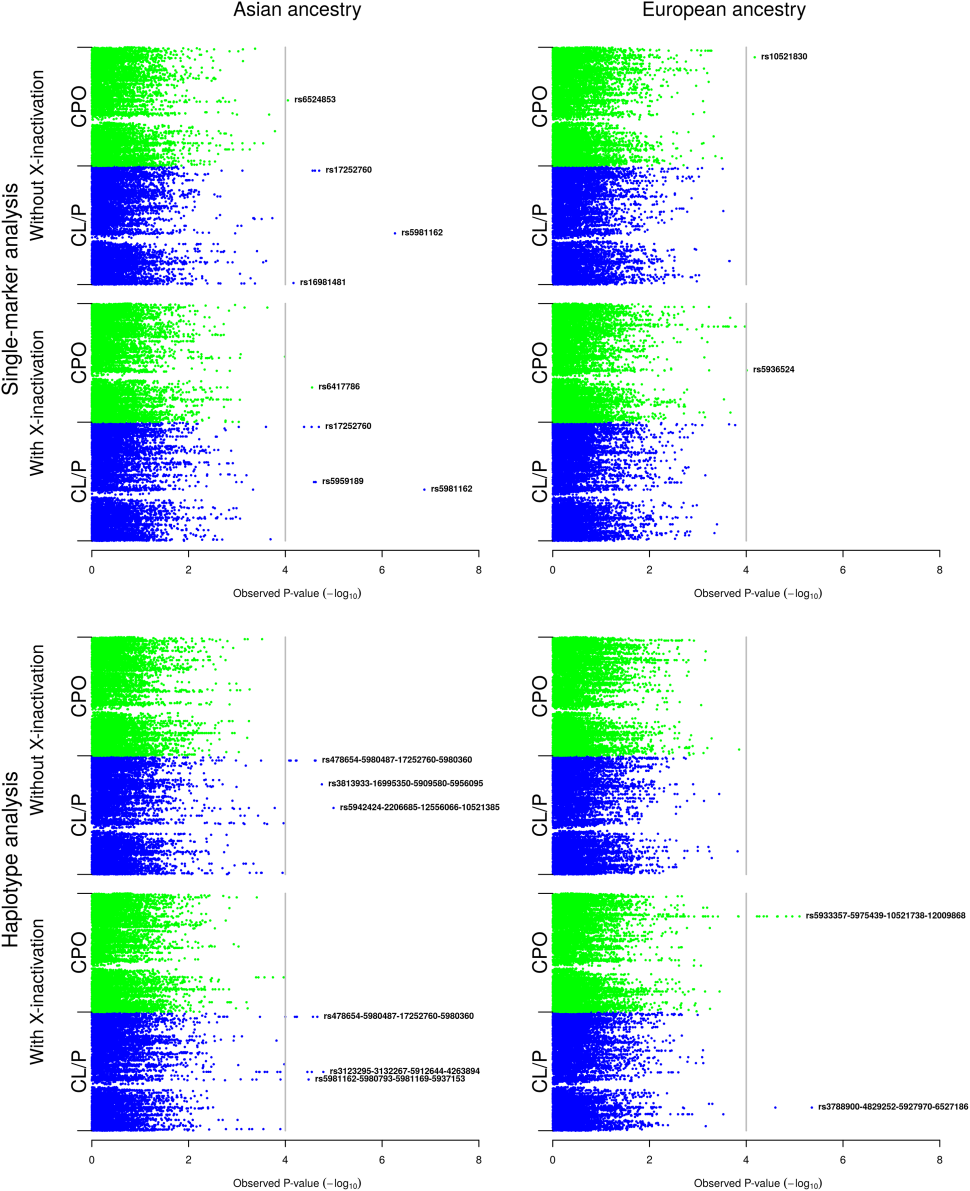
Single-marker and haplotype analyses in the Asian and European samples *without* stratification by child’s sex. The Manhattan plots show the single-marker and haplotype analyses based on the model without and with X-inactivation in females, respectively. The vertical line represents the false discovery rate (FDR) cut-off of 0.1 for declaring statistical significance.

**Fig 3:**
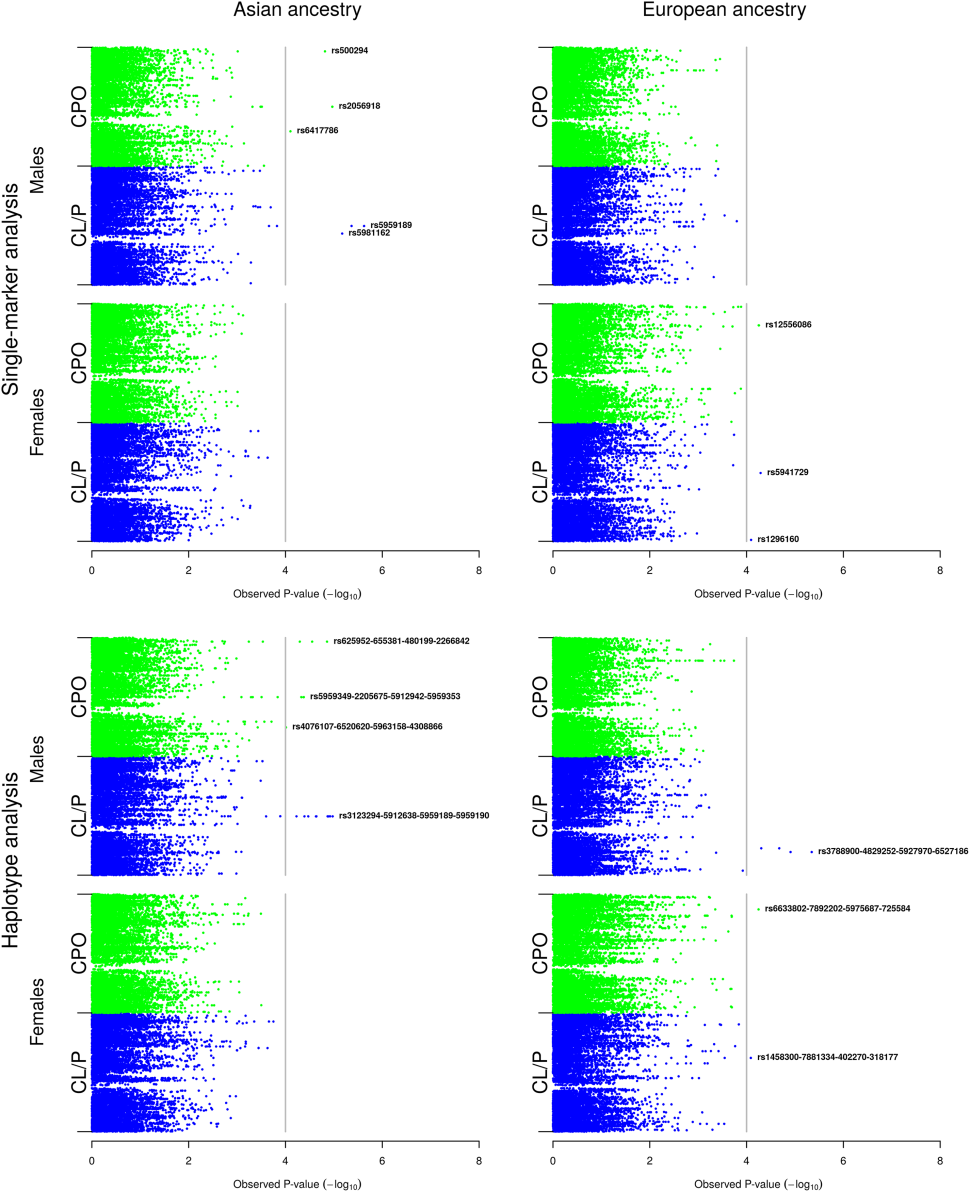
Single-marker and haplotype analyses in the Asian and European samples *with* stratification by child’s sex. The Manhattan plots show the single-marker and haplotype analyses in males and females respectively. The vertical line represents the false discovery rate (FDR) cut-off of 0.1 for declaring statistical significance.

The combination of two isolated cleft categories (CPO and CL/P), two ethnicities (Asian and European), two statistical models (with and without X-inactivation), single-marker and haplotype analyses, and analyses stratified by child’s sex generated a large amount of results. For clarity, we first present the results of the analyses without stratification by child’s sex in the Asian and European samples respectively, starting with single-marker, then haplotype analyses (Fig 2). Next, we present the results of the sex-stratified analyses (Fig 3). Detailed information on all SNPs and haplotypes that generated q-values ≤0.1 are provided in the online supplementary table S1 Table, along with their relative risks and 95% confidence intervals. We also searched for the chromosomal band locations of all the lead SNPs and haplotypes to discern patterns that would otherwise be missed when only looking at SNP IDs. Note that the Manhattan plots for the single-marker and haplotype analyses only show the lead SNPs and haplotypes. The full list of SNPs and haplotypes lying above the FDR line of ≤0.1 in Figs 2 and 3, but trailing behind the lead SNPs and haplotypes, is provided in S1 Table.

**Fig 4:**
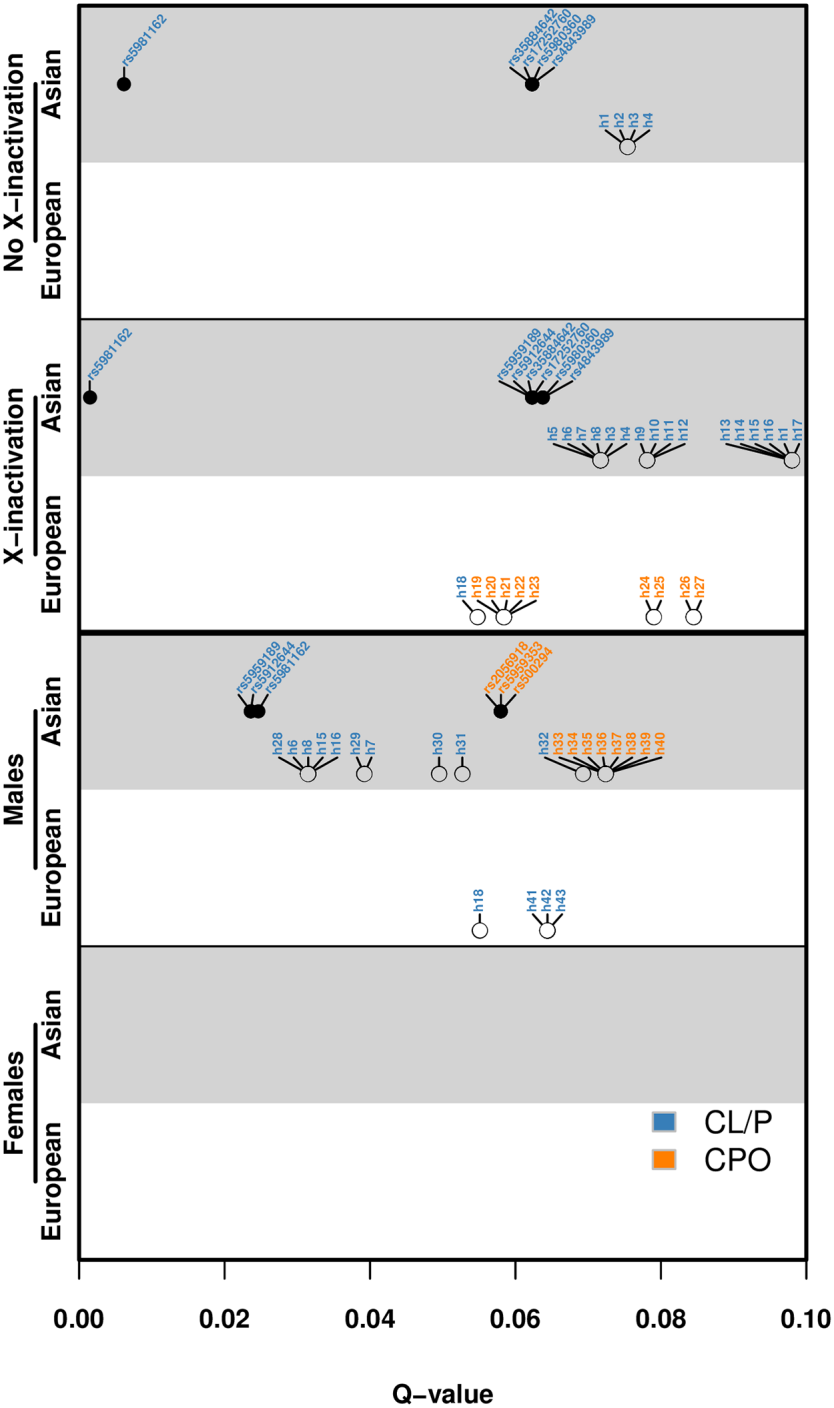
Q-value plot for all analyses generating q-values ≤0.1. The q-value plot summarizes the results for all the SNPs and haplotypes generating q-values ≤0.1. The coding of the haplotypes (h1-h43) is provided in Table 3.

**Fig 5:**
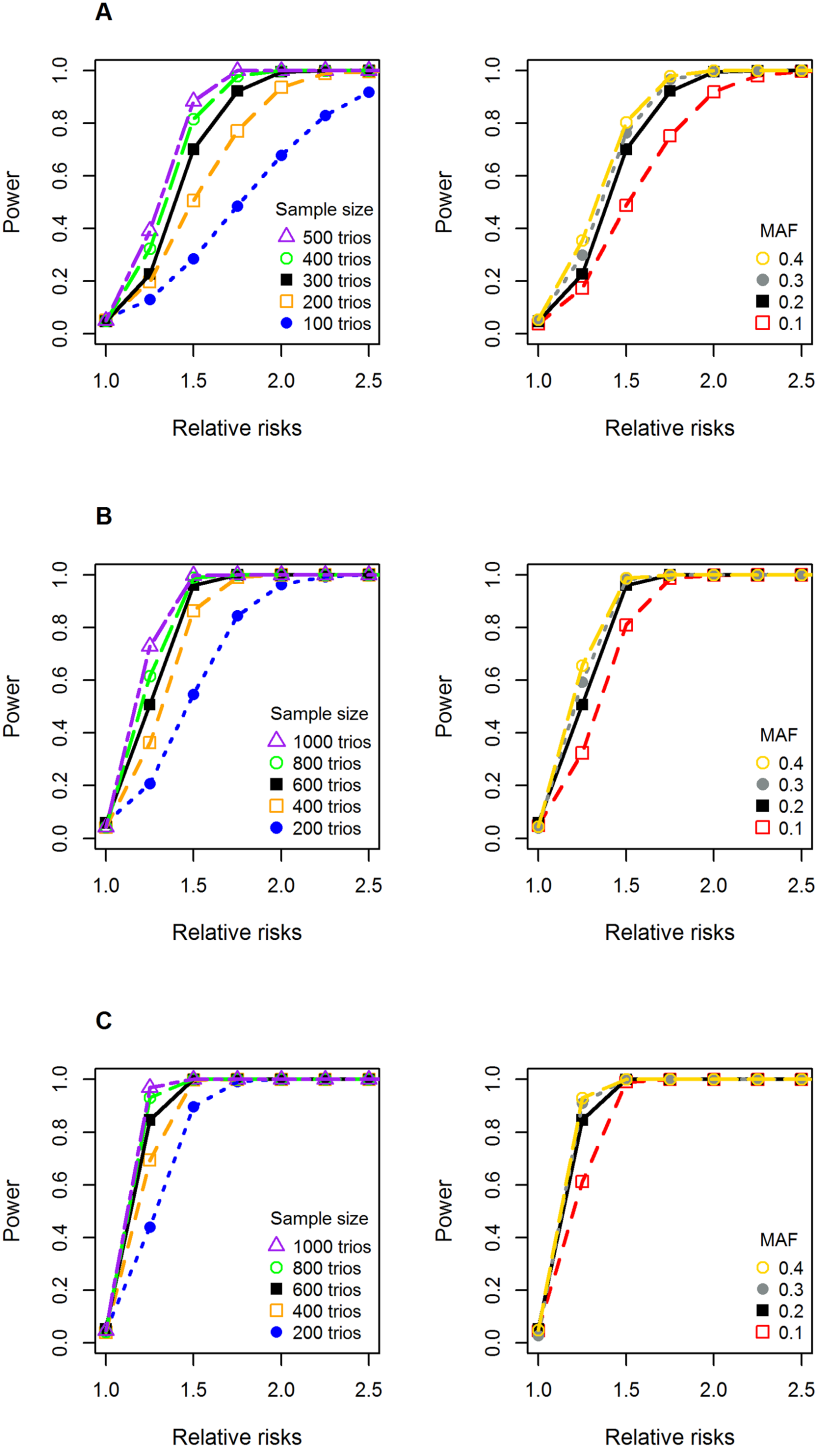
Single-SNP power calculations for different statistical models for X-linked markers. **Panel A** shows the simulated power for the sex-stratified model, **Panel B** shows the power for the model without X-inactivation in females, and **Panel C** shows the power for the model with X-inactivation. The plots on the left-hand side display power simulations with increasing relative risks and varying sample sizes of case-parent triads, assuming a SNP minor allele frequency (MAF) of 0.2. The plots on the right-hand side show the simulated power with increasing relative risks and MAFs, assuming 600 case-parent triads in the study population (300 case-parent triads for the sex-stratified model). A significance level of 0.05 was used.

In our multiplicative models, the relative risk with two copies of a variant allele is equal to the squared of the relative risk with one copy (see Fig 1). We have therefore omitted any mention of relative risks with two copies in this paper to avoid redundancy. The q-value plot in Fig 4 provides a quick visual overview of all the above analyses, and due to space limitations, the coding of the haplotypes and their chromosomal band locations are provided separately in Table 2. Fig 5 shows the results of the power simulations for different statistical models, based on sample sizes reflecting those available in the current GWAS dataset.

**Table 2:** Coding of the haplotypes used in Fig 4.

Haplotype	Chromosomal band location ^a^	Haplotype coding
rs5942424-rs2206685-rs12556066-rs10521385	Xq21.3	h1
rs3813933-rs16995350-rs5909580-rs5956095	Xq24	h2
rs478654-rs5980487-rs17252760-rs5980360	Xq28	h3
rs5980487-rs17252760-rs5980360-rs4843989	Xq28	h4
rs5981162-rs5980793-rs5981169-rs5937153	Xq13	h5
rs5959189-rs5959190-rs3123295-rs3132267	Xq21.1	h6
rs5959190-rs3123295-rs3132267-rs5912644	Xq21.1	h7
rs3123295-rs3132267-rs5912644-rs4263894	Xq21.1	h8
rs648923-rs584058-rs35884642-rs693616	Xq28	h9
rs584058-rs35884642-rs693616-rs478654	Xq28	h10
rs35884642-rs693616-rs478654-rs5980487	Xq28	h11
rs693616-rs478654-rs5980487-rs17252760	Xq28	h12
rs1918245-rs16981481-rs1527126-rs5915821	Xp22.3	h13
rs5980788-rs5981162-rs5980793-rs5981169	Xq13	h14
rs5912644-rs4263894-rs5912181-rs17324447	Xq21.1	h15
rs4263894-rs5912181-rs17324447-rs12851882	Xq21.1	h16
rs17252760-rs5980360-rs4843989-rs12013571	Xq28	h17
rs3788900-rs4829252-rs5927970-rs6527186	Xp21.1	h18
rs639-rs7883922-rs5933188-rs5977810	Xq25	h19
rs10521737-rs5930628-rs5933357-rs5975439	Xq25	h20
rs5930628-rs5933357-rs5975439-rs10521738	Xq25	h21
rs5933357-rs5975439-rs10521738-rs12009868	Xq25	h22
rs5975439-rs10521738-rs12009868-rs5978005	Xq25	h23
rs12009868-rs5978005-rs2154173-rs5930646	Xq25	h24
rs5978005-rs2154173-rs5930646-rs1936831	Xq25	h25
rs10521738-rs12009868-rs5978005-rs2154173	Xq25	h26
rs2154173-rs5930646-rs1936831-rs5975460	Xq25	h27
rs3123294-rs5912638-rs5959189-rs5959190	Xq21.1	h28
rs4573413-rs3123294-rs5912638-rs5959189	Xq21.1	h29
rs3132267-rs5912644-rs4263894-rs5912181	Xq21.1	h30
rs5912638-rs5959189-rs5959190-rs3123295	Xq21.1	h31
rs5912181-rs17324447-rs12851882-rs4313292	Xq21.1	h32
rs1751097-rs2056918-rs5912919-rs1474563	Xq21.1	h33
rs2056918-rs5912919-rs1474563-rs5959348	Xq21.1	h34
rs5912919-rs1474563-rs5959348-rs5959349	Xq21.1	h35
rs5959348-rs5959349-rs2205675-rs5912942	Xq21.1	h36
rs5959349-rs2205675-rs5912942-rs5959353	Xq21.1	h37
rs500294-rs2283746-rs625952-rs655381	Xq28	h38
rs625952-rs655381-rs480199-rs2266842	Xq28	h39
rs655381-rs480199-rs2266842-rs109389	Xq28	h40
rs3788899-rs3788900-rs4829252-rs5927970	Xp21.1	h41
rs5917959-rs5963896-rs12353703-rs5917507	Xp21.1	h42
rs5963896-rs12353703-rs5917507-rs5917197	Xp21.1	h43

^a^ Chromosomal band location was determined using NCBI’s Variation Viewer Tool available at https://www.ncbi.nlm.nih.gov/variation/view/.

### A. Single-marker and haplotype analyses without stratification by child’s sex

Fig 2 shows the Manhattan plots for the Asian and European analyses without stratifying by child’s sex. The upper half of the figure shows the single-marker analyses for the two X-inactivation models, and the bottom half shows the corresponding sliding-window haplotype analyses.

#### a) Asian sample

We observed a larger number of associations in the Asian versus the European sample (Fig 2). Both X-inactivation models identified strong associations between CL/P and the variant alleles at rs17252760 (Xq28) and rs5981162 (Xq13) (Fig 2 and S1 Table). These SNPs had q-values of 0.06 and <0.01 respectively, which are well below the cut-off of 0.1 we have used to assess statistical significance. In both X-inactivation models, the variant allele at rs17252760 increased the risk of CL/P, whereas the variant allele at rs5981162 had the opposite effect (S1 Table).

According to the 1000 Genomes browser (https://www.ncbi.nlm.nih.gov/variation/tools/1000genomes/), rs17252760 (Xq28) is located ~9.4 kb from the ‘Melanoma antigen family A, 9B’ (*MAGEA9B*) gene and rs5981162 (Xq13) is located ~62 kb from ‘Praja ring finger 1’ (*PJA1*). In addition to the above associations, the variant allele at rs5959189 (Xq21.1) had a protective effect on CL/P risk in the model with X-inactivation. This SNP is located in 'Lysophosphatidic acid receptor 4' (*LPAR4*). In the CPO category, we found associations with rs6524853 (Xq21.3) in the model without X-inactivation and with rs6417786 (Xp11.3) in the model with X-inactivation. rs6524853 is located ~40.3 kb from ‘Kelch-like family member 4’ (*KLHL4*) and rs6417786 is located in ‘Zinc finger protein 157’ (*ZNF157*).

In the haplotype analyses (lower half of Fig 2), no associations were observed with CPO in either X-inactivation models. In CL/P, rs17252760 (Xq28) and rs5981162 (Xq28) that were identified in the single-marker analyses above also appeared to be driving several of the associations here. The haplotypes generating q-values below 0.1 had RR_*SD*_ between 1.8–1.9 in the model without X-inactivation (S1 Table). In the model with X-inactivation, there were two distinct sets of haplotypes with opposite effects: the first was associated with an elevated risk of CL/P (RR_*SD*_ between 1.4-1-5), whereas the second was associated with a lower risk of CL/P (RR_*SD*_ between 0.6–0.7) (S1 Table).

Several of the genes identified in the haplotype analyses were also identified in the single-marker analyses (e.g. *MAGEA9B*, *PJA1*, and *LPAR4*). In the model without X-inactivation, the last SNPs in rs5942424-rs2206685-rs12556066-rs10521385 (Xq21.3) is located ~109 kb from ‘CPX chromosome region, candidate 1’ (*CPXCR1*)—one among several genes associated with X-linked cleft palate (CPX) [27]. SNPs in the haplotype rs3813933-rs16995350-rs5909580-rs5956095 (Xq24) are located in ‘LON peptidase N-terminal domain and ring finger 3’ (*LONRF3*). In the model with X-inactivation, three haplotypes were associated with CL/P: i) rs478654-rs5980487-rs17252760-rs5980360 (Xq28), ii) rs3123295-rs3132267-rs5912644-rs4263894 (Xq21.1), iii) rs5981162-rs5980793-rs5981169-rs5937153 (Xq13). The SNPs in haplotype i) lie near *MAGEA9B*; those in haplotype ii) are located near *LPAR4*, and SNPs in haplotype iii) lie near *PJA1*.

#### b) European sample

As noted above, there were distinctly fewer associations in the European versus the Asian sample. In the single-marker analyses (upper half of Fig 2), the variant allele at rs10521830 (Xq27) was associated with CPO in the model without X-inactivation. There were no associations with CL/P. In the model with X-inactivation, the variant allele at rs5936524 (Xq13) was associated with CPO. Again, there were no associations with CL/P. rs10521830 is not located near any known gene within 20 kb, whereas rs5936524 is located in ‘Ectodysplasin A’ (*EDA*).

In the haplotype analyses (lower half of Fig 2), no associations were detected in the model without X-inactivation. In the model with X-inactivation, rs5933357-rs5975439-rs10521738-rs12009868 (Xq25) was associated with CPO and rs3788900-rs4829252-rs5927970-rs6527186 (Xp21.1) was associated with CL/P. SNPs in the first haplotype are located in ‘DDB1 and CUL4 associated factor 12 like 2’ (*DCAF12L2*), and SNPs in the second haplotype are located in dystrophin (*DMD*)—the second largest gene in humans according to the length of the transcript and the protein product [28].

### B. Single-marker and haplotype analyses stratified by child’s sex

Fig 3 displays the Manhattan plots for the Asian and European analyses, this time with stratification by child’s sex. As before, the upper half of the figure shows the single-marker analyses for males and females, and the bottom half shows the corresponding sliding-window haplotype analyses.

#### a) Asian sample

The variant alleles at rs500294 (Xq28), rs2056918 (Xq21.1) and rs6417786 (Xp11.3) were associated with CPO in the single-marker analyses of the male sample (upper half of Fig 3). None of the associations was observed among the females, suggesting they may be sex-specific. rs500294 is located in ‘Mastermind-like domain containing 1’ (*MAMLD1*), rs2056918 is ~6 kb from ‘Integral membrane protein 2A’ (*ITM2A*), and rs6417786 is located in *ZNF157*. *ITM2A* has previously been identified as a contributor to sexual dimorphism in human height [11]. In addition to being located near *ITM2A*, rs2056918 is also ~641 kb from ‘T-box 22’ (*TBX22*). Although this is far in the context of linkage disequilibrium, the proximity to *TBX22* is notable because mutations in this gene cause the inherited X-linked disorder ‘Cleft palate with ankyloglossia’ (CPX). In the CL/P category, we found associations with the variant allele at rs5981162 (Xq13; q = 0.024), similar to what was observed in the analyses without stratification by child’s sex, and with the variant allele at rs5959189 (Xq21.1; q = 0.024). As with CPO, the associations with CL/P were only detected among the males. rs5981162 is located ~62 kb from ‘Praja ring finger 1’ (*PJA1*) and rs5959189 is located in *LPAR4*.

In the haplotype analyses (lower half of Fig 3), there were several associations among the males in both cleft categories, but none among the females. Three lead haplotypes were associated with CPO: i) rs625952-rs655381-rs480199-rs2266842 (Xq28), ii) rs5959349-rs2205675-rs5912942-rs5959353 (Xq21.1) and iii) rs4076107-rs6520620-rs5963158-rs4308866 (Xp11.4). SNPs in haplotype i) are located in *MAMLD1*, SNPs in haplotype ii) are not located within 20 kb of any gene, whereas all the SNPs in haplotype iii) lie in ‘BCL6 co-repressor’ (*BCOR*). In CL/P, we found an association with rs3123294-rs5912638-rs5959189-rs5959190 (Xq21.1), the first three SNPs of which are located in *LPAR4*.

#### b) European sample

There were no associations among the males in the single-marker analyses (upper half of Fig 3). In the female sample, the variant allele at rs12556086 (Xq25) was associated with CPO, and those at rs5941729 (Xq21.3) and rs1296160 (Xp22.3) were associated with CL/P. These SNPs are not located within 20 kb of any known gene.

In the haplotype analyses (lower half of Fig 3), rs3788900-rs4829252-rs5927970-rs6527186 (Xp21.1; q = 0.055) was the lead haplotype associated with CL/P among the males. All the SNPs in this haplotype are located in *DMD*. In the female CL/P sample (Fig 3), we identified associations with the following two lead haplotypes: i) rs6633802-rs7892202-rs5975687-rs725584 (Xq26) in CPO and ii) rs1458300-rs7881334-rs402270-rs318177 (Xq21.3) in CL/P. SNPs in the first haplotype i) are located in and near the gene for ‘Four and a half LIM domains 1’ (*FHL1*). SNPs in the second haplotype ii) are not located close to any gene within 20 kb.

### Ethnic- and sex-specificity

None of the associated SNPs or haplotypes was in common across the two ethnicities. Our analyses stratified by child’s sex showed strong evidence of sex-specificity for several of the associated SNPs and haplotypes (Fig 3). In the Asian sample, in particular, we identified associations only among the males in both single-marker and haplotype analyses, and associations that were originally identified in the analyses without sex-stratification reappeared exclusively among the males. Although less striking, this sex specificity was also apparent in the European sample.

### Power simulations

We performed statistical power simulations on 1000 replicates of data for a single SNP to investigate the *a priori* power of detecting the effects of X-linked SNPs using the different model parameterizations in this paper (Fig 5). All the power analyses were performed in HAPLIN [29]. In power simulations for the statistical model with stratification by child’s sex, the sample sizes were simply halved and the baseline risk set equal to 1. Our results show that, for single-marker analyses, there was sufficient power (at a 5% significance level) for most of the sample sizes tested here, given a relative risk >1.5 and a minor allele frequency >0.2.

## Discussion

The flexible estimation capabilities in HAPLIN enabled an exploration of alternative patterns of effects due to X-inactivation and child’s sex. We found a larger number of associations in the Asian versus the European sample in both single-marker and haplotype analyses. Our analyses also uncovered strong evidence of sex-specificity among the detected associations, and confirmed the considerable genetic heterogeneity previously reported in these two ethnically diverse samples [17]. Table 3 provides a synopsis of all the genes in which associations were found, the statistical model and sex in which they were identified, and how our current findings relate to those of four previous studies [6–9]. Of the 16 genes listed in Table 3, four were only identified by the model without X-inactivation (*CPXCR1*, *EDA*, *KLHL4*, and *LONRF3*); four were only identified by the model with X-inactivation (*DCAF12L2*, *DMD*, *LPAR4* and *ZNF157*); four more were only identified in the Asian male sample (*BCOR*, *ITM2A*, *MAMLD1* and *TBX22*), and one was only identified in the European female sample (*FHL1*). Furthermore, *EFNB1/PJA1* and *MAGEA9B* were the only genes identified by both X-inactivation models, and *DMD* was identified both in the European male sample and by the model assuming X-inactivation.

**Table 3:** Synopsis of all genes with which associations were identified in this study.

Gene ^a^	Location	Full name	Identified in model/sex	Comments ^b^	Key references ^c^
*BCOR*	Xp11.4	BCL6 corepressor	Asian males	BCOR is a corepressor of BCL6, a transcription repressor required for germinal center formation. Mutations in *BCOR* cause the oculo-facio-cardio-dental (OFCD) syndrome. Patients with OFCD have several facial abnormalities, including cleft palate.	[45–47]
*CPXCR1*	Xq21.31	CPX chromosome region, candidate 1	No X-inactivation	*CPXCR1* is localized to a critical region on the X chromosome associated with an X-linked cleft palate (CPX) disorder. However, mutation screening has shown that variants in this gene are unlikely to be the cause of CPX.	[27]
*DCAF12L2*	Xq25	DDB1 and CUL4 associated factor 12 like 2	X-inactivation	*DCAF12L2* encodes a member of the WD-repeat protein family known to be involved in various cellular processes, including cell cycle progression, signal transduction, apoptosis and gene regulation. Not previously associated with clefts.	NA
***DMD***	Xp21.2	Dystrophin	X-inactivation, European males	Dystrophin is a component of the dystrophin-glycoprotein complex that provides stability by bridging the cytoskeleton of the muscle to the extracellular matrix. Mutations in *DMD* cause Duchenne (DMD) and Becker (BMD) muscular dystrophies. This gene was identified by all four studies (Patel *et al*. (2013), Wise *et al*. (2015), Fonseca *et al*. (2015), and the current study).	[6–8]
*EDA*	Xq12-q13.1	Ectodysplasin A	No X-inactivation	*EDA* encodes a protein belonging to the tumor necrosis factor family. Mutations in *EDA* cause X-linked hypohidrotic ectodermal dysplasia. A contiguous deletion containing *EFNB1*, *OPHN1*, *PJA1* and *EDA* was reported in patients with craniofrontonasal syndrome (CFNS). Goyal *et al*. (2015) reported a case with X-linked hypohidrotic ectodermal dysplasia with cleft palate. The gene for CFNS is *EFNB1*, but because *EFNB1* lies close to *EDA* and *PJA1*, associations with these genes might be the result of linkage disequilibrium.	[35, 36, 48]
*EFNB1*	Xq13.1	Ephrin B1	Like *PJA1*, both X-inactivation models	*EFNB1* encodes ephrin-B1, a transmembrane ligand for Eph receptor tyrosine kinases. Mutations in *EFNB1* cause craniofrontonasal syndrome, which features cleft lip and palate among other clinical features.	[33, 34, 49]
*FHL1*	Xq26	Four and a half LIM domains 1	European females	*FHL1* belongs to the four-and-a-half-LIM-only protein family. Mutations in this gene have been found in patients with Emery-Dreifuss muscular dystrophy (EDMD). Besides *DMD*, this is the second gene HAPLIN identified with a link to muscular dystrophy.	[37]
*ITM2A*	Xq21.1	Integral membrane protein 2A	Asian males	*ITM2A* is involved in early cartilage development and has been shown to be a potential contributor to sexual dimorphism in human height. Expression data show that *ITM2A* escapes from X-chromosome inactivation in the majority of women. Not previously associated with clefts.	[11, 50]
*KLHL4*	Xq21.3	Kelch-like family member 4	No X-inactivation	*KLHL4* is a member of the kelch family of proteins. These proteins are characterized by kelch repeat motifs and a POZ/BTB protein-binding domain. *KLHL4* maps within the critical region for ‘X-linked cleft palate and ankyloglossia’ (CPX). Like *CPXCR1* described above, *KLHL4* is also widely expressed in fetal tissues, including the tongue, palate and mandible—consistent with the CPX phenotypes.	[27, 42]
*LONRF3*	Xq24	LON peptidase N-terminal domain and ring finger 3	No X-inactivation	*LONRF3* encodes a protein containing a RING finger domain that is involved in protein-protein and protein-DNA interactions. Not previously associated with clefts.	NA
*LPAR4*	Xq21.1	Lysophosphatidic acid receptor 4	X-inactivation	*LPAR4* encodes a member of the lysophosphatidic acid receptor family. Not previously associated with clefts.	[51]
***MAGEA9B***	Xq28	MAGE family member A9B	No X-inactivation	*MAGEA9B* is a duplication of the *MAGEA9* gene on chromosome Xq28. Not previously associated with clefts.	NA
*MAMLD1*	Xq28	Mastermind-like domain containing 1	Asian males	*MAMLD1* encodes a mastermind-like domain containing protein. Mutations in *MAMLD1* cause X-linked hypospadias type 2 (HYSP2). Not previously associated with clefts.	[52]
***PJA1*** ^d^	Xq13.1	Praja ring finger 1, E3 ubiquitin protein ligase	Both X-inactivation models	*PJA1* encodes an enzyme carrying a RING-H2 motif. Deletion of a DNA segment involving *EFNB1*, *OPHN1*, *PJA1* and *EDA* has been associated with craniofrontonasal syndrome (CFNS). CFNS is caused by mutations in *EFNB1*, but because this gene lies close to *EDA* and *PJA1*, associations with these genes might be due to linkage disequilibrium. *PJA1* was also identified in Patel *et al*. (2013) and Wise *et al*. (2015).	[33, 36, 53]
*TBX22*	Xq21.1	T-box 22	Asian males	TBX22 belongs to a conserved family of transcription factors that share a DNA-binding domain called the T-box. Mutations in *TBX22* underlie the inherited X-linked disorder, cleft palate with ankyloglossia (CPX).	[43]
*ZNF157*	Xp11.2	Zinc finger protein 157	X-inactivation	Encodes a member of the Zn-finger family of transcription factors. Not previously associated with clefts.	[54]

^a^ Genes that were in common with those in Patel *et al*. (2013), Fonseca *et al*. (2015), and Wise *et al*. (2015, 2016) are emboldened.

^b^ Information about the genes was collated from Entrez Gene (http://www.ncbi.nlm.nih.gov/gene/) and the indicated references.

^c^ NA: Not applicable because no relevant references are available for the gene.

^d^ This gene lies upstream of ‘Ephrin-B1’ (*EFNB1*; Xq12) and downstream of ‘Ectodysplasin A’ (*EDA*; Xq13.1).

### Comparisons with previous studies

Our research group was among the first to investigate genetic associations between X-linked SNPs and isolated orofacial clefts, initially in a study that targeted candidate genes [5]. We are aware of only four studies to date that have tested for associations between orofacial clefts and SNPs across the entire X-chromosome [6–9]. Three of these [6, 7, 9] were based on the same GWAS dataset as in the current study, enabling a comparison of findings across different methods. Patel *et al*. (2013) used ‘Family-Based Association Tests’ (FBAT) [30, 31] and Wise and co-workers used their own methods, PIX-LRT [7] and PIX-HAP [9]. Fonseca *et al*. [8] used X-TDT [15] to analyze 23,344 SNPs in 27 Argentinian mother-child dyads of orofacial clefts.

Among our findings, only *DMD* and *EFNB1/PJA1* overlapped with those of Patel *et al*. (2013). Given both studies used the same GWAS dataset, we anticipated more commonality across these two studies irrespective of methodological differences. We did however detect associations with the same SNPs as in Patel *et al*. (2013) for *EFNB1*/*PJA1*, but not for *DMD*. The most strongly associated SNP (‘lead SNP’) may differ across studies owing to inherent methodological differences and varying degrees of linkage disequilibrium between neighboring SNPs. Different methods may pick up associations with the same gene or locus, but not necessarily with the same lead SNP. Haplotype analyses, like the ones conducted here, are valuable in these situations. Our haplotype analyses differed from those in Wise *et al*. (2016) and Patel *et al*. (2013) in that they were based on a sliding-window approach using contiguous SNPs to reconstruct all haplotype combinations, as opposed to studying combinations of SNPs. This could account for some of the disparity observed in the findings across these studies. Moreover, differences in SNP-hits between these studies may also be attributed to differences in data-cleaning/quality-control steps and in the use of incomplete observations. We have not attempted a systematic comparison of the different methods.

**Fig 6:**
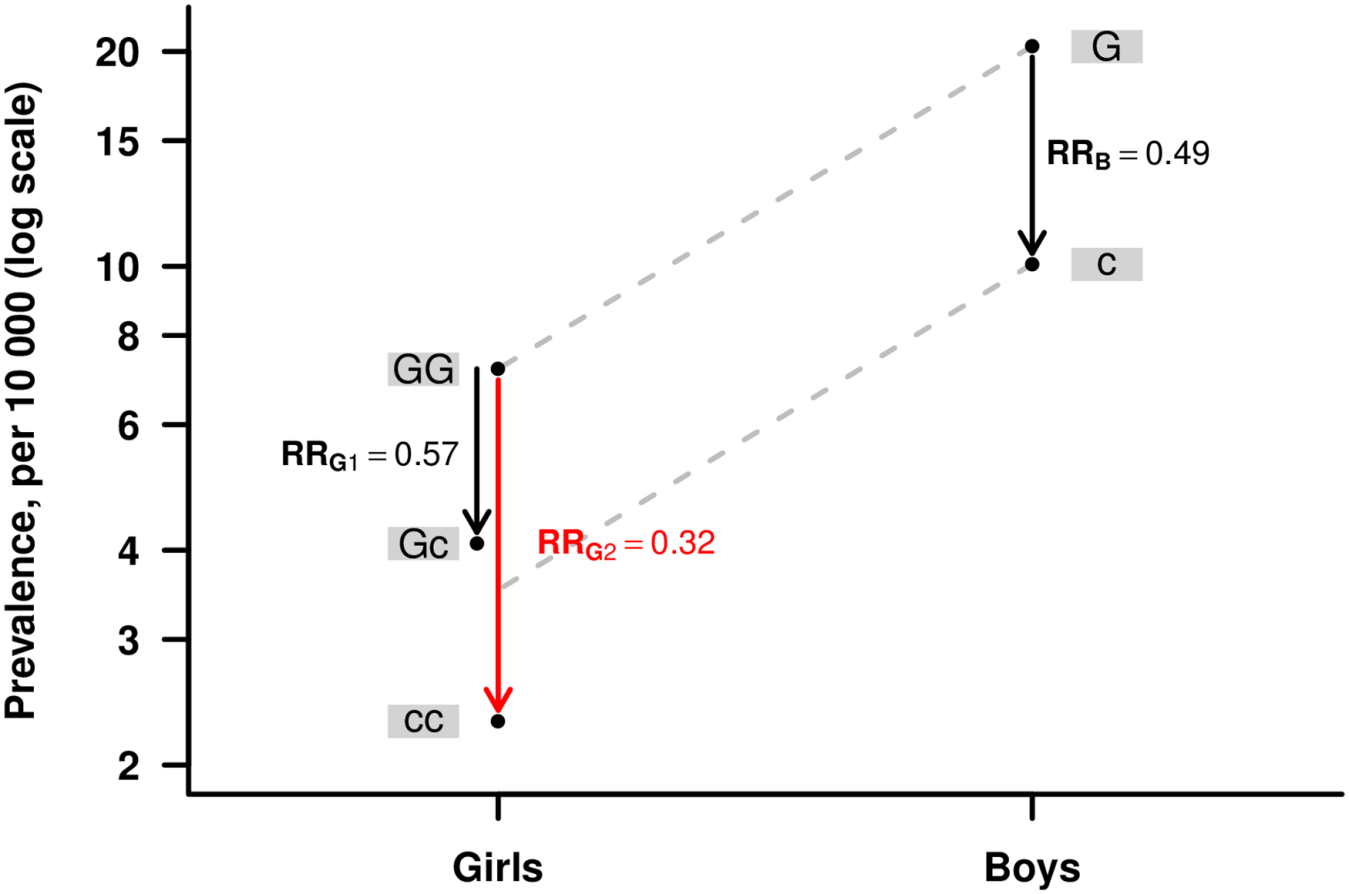
Effects of rs5981162 when estimated separately for boys and girls under the multiplicative model. Left black arrow: relative (reduction in) risk for girls inheriting a single dose of the allele “c”, RRG1 = 0.57. Red arrow: relative (reduction in) risk for girls inheriting a double dose of the allele “c”, RRG2 = RRG1·RRG1 = 0.32. Right black arrow: relative (reduction in) risk for boys inheriting a single dose of the allele “c”, RRB = 0.49. Grey dashed line shows that the effect for boys falls in between what would be expected from X-inactivation and no X-inactivation in girls.

Wise *et al*. (2015) used PIX-LRT to analyze X-chromosome SNPs and, like Patel *et al*. (2013), analyzed the same GWAS dataset as here. Their study found no statistically significant associations with CPO, but associations with the following genes were identified in CL/P: *EFNB1*/*PJA1*, *MAP7D3*, *FUNDC1*, *DUSP21*, and *DMD* [7]. Again, *EFNB1*/*PJA1* and *DMD* are in common with our findings. For *PJA1*, we identified a strong association with the same lead SNP (rs5981162; Xq13) as in Wise *et al*. (2015) and Patel *et al*. (2013), in both X-inactivation models. The association with the “c”-allele at rs5981162 is particularly interesting because it illustrates the importance of exploring different X-inactivation models. The effect for boys fell in between what would be expected from X-inactivation and no X-inactivation in girls (Fig 6). (Note that the prevalence for boys and girls was computed using Norwegian data provided in Table 3 in Harville *et al*. [32].)

If we set aside the study by Fonseca *et al*. (2015), *EFNB1*/*PJA1* and *DMD* are the only two genes in common across three of the above studies and methods (Table 3). The Fonseca *et al*. (2015) study is particularly insightful because it still managed to replicate the association with *DMD* despite analyzing only a modest number (*N* = 27) of mother-child dyads of orofacial clefts in a different study population. That study also identified associations with three other loci (at Xp11.4, Xp22.31 and Xp11.23) besides the one containing *DMD* (Xp21.1). Only the locus at Xp11.4 remained significant after correcting for multiple testing [8]. However, our finding of a significant association with a locus at Xp22.3, notably with haplotype rs1918245-rs16981481-rs1527126-rs5915821 in the Asian CL/P sample (‘h13’ in Fig 4), corroborates the findings in Fonseca *et al*. (2015).

Prior to Patel *et al*. (2013), neither *EFNB1*/*PJA1* nor *DMD* had previously been linked with orofacial clefts in a genetic association study. As also noted by Wise *et al*. [7], *PJA1* lies ~318.6 kb upstream of ‘Ephrin-B1’ (*EFNB1*) and ~450.5 kb downstream of ‘Ectodysplasin A’ (*EDA*)—between two genes already implicated in orofacial clefts. Mutations in *EFNB1* cause craniofrontonasal syndrome, which features cleft lip and palate among other clinical manifestations [33, 34]. *EDA* is involved in X-linked ‘hypohidrotic ectodermal dysplasia’ (HED) [35]. Furthermore, a contiguous deletion of a region containing *EFNB1*, ‘Oligophrenin 1’ (*OPHN1*), *PJA1* and *EDA* was reported in patients with craniofrontonasal syndrome [36]. It is thus likely that the associations with *PJA1* are due to linkage disequilibrium with SNPs in *EFNB1* or *EDA*, but this needs to be verified in other cleft populations.

The strong association with *DMD* in our data was all the more striking because yet another gene implicated in muscular dystrophy—*FHL1*—was identified in the European female sample (Fig 3). Mutations in *FHL1* have been observed in patients with ‘Emery-Dreifuss muscular dystrophy’ (EDMD) [37]. The link between muscular dystrophy and CL/P is not unprecedented, however, as illustrated by the Walker-Warburg syndrome—a rare autosomal recessive disorder characterized by congenital muscular dystrophy, brain and eye malformations, and CL/P [38–41]. Future efforts in other well-powered cleft samples should help to decipher the links between muscular dystrophy and isolated clefts.

In addition to *EFNB1/PJA1* and *DMD*, associations with *KLHL4*, *CPXCR1*, *TBX22*, and *BCOR* were noteworthy because of their roles in clefting syndromes. *KLHL4* maps to the critical region for the semi-dominant disorder ‘X-linked cleft palate and ankyloglossia’ (CPX) [27]. *KLHL4* is expressed in several fetal tissues, including the tongue, palate and mandible—consistent with the CPX phenotypes [42]. *CPXCR1* is one among several genes identified in the critical region for X-linked cleft palate [27]. Mutations in the T-box containing transcription factor *TBX22* cause ‘X-linked cleft palate including ankyloglossia’ (CPX) [43]. Finally, mutations in *BCOR* cause the ‘Oculo-facio-cardio-dental’ (OFCD) syndrome, which features palatal anomalies, septate nasal tip, high nasal bridge and midface hypoplasia, among several craniofacial abnormalities [44–46]. Given the strong connections with clefting syndromes, elucidating the contributions of *KLHL4*, *EFNB1/PJA1*, *CPXCR1*, *TBX22* and *BCOR* to isolated forms of clefting offers exciting new avenues for future research. Furthermore, associations with the remaining genes in Table 3 (e.g. *DCAF12L2*, *ITM2A*, *LONRF3*, *LPAR4*, *MAGEA9B*, *MAMLD1* and *ZNF157*) are novel and warrant further investigations.

### Strengths and weaknesses

Our study was based on the case-parent triad design where the strict independence of marker genotype and observed phenotype is tested and bias due to population stratification is effectively circumvented [20]. Such biases may arise in conventional case-control settings if marker allele frequencies vary across unrecognized subpopulations in the case and control groups. Also, our study was based on the largest available collection of Asian and European case-parent triads to date, providing an excellent opportunity to explore ethnic- and sex-specific differences for each of the identified associations. Having GWAS triad-data on these two major ethnicities enabled an agnostic approach to association analysis, as opposed to the hypothesis-driven candidate-gene approach. Notably, our previous work [5] on cleft candidate genes in 562 Norwegian and 235 Danish case-parent triads pointed to an association between isolated CL/P and the ‘Oral-facial-digital syndrome 1’ (*OFD1*) gene in the Danish but not the Norwegian triads. This association was not clearly replicated in our current chromosome-wide analysis. The two SNPs analyzed in the previous candidate analysis (rs2285635 and rs2283707) were not available in the current GWAS dataset. Only one SNP, rs6527959, represented *OFD1* in the GWAS dataset, and this SNP only achieved moderate significance for isolated cleft lip only (CLO) in European males (p-value 0.0091), hardly convincing in light of the number of models, endpoints and ethnicities tested. By studying a smaller number of SNPs in a few candidate genes (48 SNPs in 18 genes), our previous candidate-gene analysis might have been more focused and less influenced by the limitations of multiple testing. Still, the association with *OFD1* could have been a false positive, as hinted by the absence of replication in the closely-related Norwegian CL/P triads.

### Power considerations

Statistical power is constrained by several factors, one of which is the frequency of the variant allele, which may differ considerably among ethnic groups of diverse ancestry. The gap in power, depending on allele frequencies, is demonstrated in Fig 5 (right-hand side). The power analyses were conducted for a single-SNP, and the difference in power due to varying allele frequencies would be even more evident in a GWAS setting. Checking the SNPs with the lowest q-values (Fig 4), the allele frequencies were slightly lower among Asians than Europeans for rs5959189 (Asians: 0.058; Europeans: 0.087) and for rs5912644 (Asians: 0.056; Europeans: 0.088), which means that the lower q-values for Asians are not due to different allele frequencies. For rs5981162 (Asians: 0.13; Europeans: 0.015), however, the very low allele frequency among Europeans would have made it hard to achieve significance, conceivably explaining some of the population difference for this SNP.

Our power simulations showed that we had sufficient power for single-SNP analyses based on the sample sizes available in our GWAS dataset. Besides allele/haplotype frequencies, the power to detect the effects of X-linked markers also depends on the parameterization model, effect size, and type of family design. The power from a full GWAS would be limited due to multiplicity, which needs to be taken into account when interpreting our findings.

## Conclusions

Our study provided further support for a role for X-linked variants in isolated clefts by confirming previously identified associations with *DMD* and *EFNB1/PJA1* and identifying new ones for further investigation. Associations with *KLHL4*, *EFNB1/PJA1*, *CPXCR1*, *TBX22*, and *BCOR* were particularly noteworthy because they are involved in syndromes that feature orofacial clefts. Our study also highlighted marked ethnic- and sex-specificity for the associations identified in the Asian and European sample. The low transferability of these associations further underscores the need to appraise whether an association identified in one ethnic group is generalizable to another. Additionally, several of the associations were confined to one particular X-inactivation model or child’s sex, emphasizing the need for flexible analytic tools that are capable of detecting and estimating such effects. The methods presented here should therefore be of wide applicability to other complex traits in which a family-based study-design is implemented.

## Supporting information

S1 Table Q-values and RR estimates. (This supplementary table is provided separately as an MS Excel file.)
(XLSX)
